# Prevalence and determinants of probable dementia in rural India: a cross-sectional study in the APCAPS cohort

**DOI:** 10.7189/jogh.16.04339

**Published:** 2026-10-19

**Authors:** Jagannadha Rao Basa, Kanokporn Pinyopornpanish, Poppy AC Mallinson, Papangkorn Inkeaw, Kanokwan Kulprachakarn, Judith Lieber, Santosh Kumar Banjara, Hemant Mahajan, Teena Dasi, Santhi Bhogadi, Srivalli Addanki, Gowri Iyer, Anoop SV Shah, Om Kurmi, Sureshkumar Kamalakannan, Sanjay Kinra, Chaisiri Angkurawaranon

**Affiliations:** 1 Research Institute for Health Sciences, Chiang Mai University, Chiang Mai, Thailand; 2 Department of Family Medicine, Faculty of Medicine, Chiang Mai University, Chiang Mai, Thailand; 3 Global Health and Chronic Conditions Research Group, Chiang Mai University, Chiang Mai, Thailand; 4 Department of Non-communicable Disease Epidemiology, London School of Hygiene and Tropical Medicine, Faculty of Epidemiology and Population Health, London, UK; 5 Data Science Research Center, Faculty of Science, Chiang Mai University, Chiang Mai, Thailand; 6 ICMR-National Institute of Nutrition, Hyderabad, Telangana, India; 7 Public Health Foundation of India, New Delhi, India; 8 Indian Institute of Public Health Hyderabad, Hyderabad, India; 9 Centre for Global Chronic Conditions, Department of Non-communicable Disease Epidemiology, Faculty of Epidemiology and Population Health, London School of Hygiene & Tropical Medicine, London, UK; 10 Coventry University, Coventry, UK; 11 Department of Social Work Education and Community Wellbeing, Northumbria University, Newcastle, UK; 12 International Center for Evidence in Disability, London School of Hygiene & Tropical Medicine, London, UK; 13 Indian Council of Medical Research, New Delhi, India

**Keywords:** probable dementia, prevalence, cardiometabolic risk, low-literary, rural India

## Abstract

**Background:**

Dementia poses a growing public health challenge in India. Despite this, epidemiological data on the condition remain largely restricted to adults >60 years of age, despite the convergence of urbanisation-driven cardiometabolic risks and socioeconomic disadvantage in rural India potentially accelerating dementia onset. We investigated the prevalence and determinants of probable dementia in a transitional rural Indian cohort of adults aged ≥45 years.

**Methods:**

In this cross-sectional study, we utilised data from the Andhra Pradesh Children and Parents Study cohort from 29 villages near Hyderabad, South India (2021–2023). We collected sociodemographic, occupational, and household asset data through face-to-face, questionnaire-based interviews. Cardiometabolic diseases (diabetes, hypertension, cardiovascular disease, and stroke) were assessed using validated methods. Dementia screening was performed using the Brief Community Screening Instrument for Dementia. We calculated prevalence estimates for dementia and used multivariable logistic regression to identify independent associations of probable dementia with sociodemographic, socioeconomic (education, occupation, household assets), and cardiometabolic factors (diabetes, hypertension, cardiac disease, and stroke).

**Results:**

The final sample included 2,163 adults aged ≥45 years. The prevalence of probable dementia (a positive screen on the BCSI-D) was 3.4% (95% confidence interval (CI) = 2.6–4.6) in the 45–59 age group and 7.4% (95% CI = 6.0–9.4) in the ≥60 age group. In the multivariable model, the odds of probable dementia were 2.2 times higher among unemployed or retired participants than among those working in agriculture or animal rearing (adjusted odds ratio (aOR) = 2.23; 95% CI = 1.26–3.95). The odds of probable dementia among literate participants were 0.5 times those among illiterate participants (aOR = 0.54; 95% CI = 0.31–0.89). The odds of probable dementia were 3.8 times higher among participants with a history of stroke than among those without (aOR = 3.77; 95% CI = 1.67–7.94).

**Conclusions:**

Our results indicate that the burden of probable dementia in rural India extends into middle age, and that the condition itself is independently associated with a history of stroke and socioeconomic disadvantages. This suggests a need to extend cognitive and vascular risk assessment to adults aged ≥45 years in rural India. However, as all findings reflect positive screening results rather than confirmed diagnoses, longitudinal studies with diagnostic confirmation are needed before population-level screening recommendations can be made.

Dementia poses an emerging public health crisis in India, where it is driven by the country’s demographic transition toward an ageing population [1]. Recent data from the Longitudinal Ageing Study (LASI) 2023 estimates the prevalence of dementia among adults aged ≥60 years in India to be 7.4%, with significantly higher rates in rural areas (8.4%) compared to urban centres (5.3%) [2]. This higher prevalence has been attributed to the lower socioeconomic status, lower education levels, and nuclear family structures present among rural populations, and is further compounded by cardiometabolic risk factors that contribute to cognitive decline and mortality [3,4].

Dementia exhibits distinct epidemiological profiles among different age groups. Research indicates that Alzheimer’s disease predominates in elderly individuals aged ≥60 years, among whom it accounts for 47% of dementia cases, while vascular and frontotemporal dementia account for 58% and 41% of cases among <60-year-olds, respectively [5]. Clinical studies report that the onset of these subtypes of dementia occurs at least 10 years earlier than Alzheimer’s disease [6], suggesting they have a different aetiology driven heavily by vascular and metabolic factors [5–8].

 Despite this distinct profile, three critical gaps remain in research on the prevalence of dementia in India. First, data on the prevalence of dementia among adults aged 45–59 years are lacking, with existing studies being dated, enrolling only adults aged ≥60 years, and relying on clinical diagnoses, rather than community-based screening [9–12]. Second, adults aged <60 years in India have high rates of comorbidities such as hypertension and diabetes, which are associated with increased dementia risk [13–17]. However, the relationship between cardiometabolic risk factors and early cognitive decline has not been adequately examined in Indian populations. Finally, the influence of socioeconomic determinants remains underexplored in India, with few studies investigating how factors such as occupation and household assets associate dementia risk in rural and largely illiterate populations [18–20].

To address these gaps, we analysed data obtained from the Andhra Pradesh Children and Parents Study (APCAPS) cohort to estimate the prevalence of probable dementia among adults aged ≥45 years in rural India. We used 45 years as the lower age threshold because evidence suggests that the burden of young-onset dementia and its associated vascular risk factors begins to increase during midlife, particularly in low- and middle-income countries [5,13,21], and because the incidence of stroke, a major contributor to vascular cognitive decline, is known to increase sharply among individuals aged 40–49 years and remains high in those aged 45–59 years [22,23]. Furthermore, the ≥45-year threshold is consistent with contemporary population-based ageing research in India, including longitudinal cohort studies that enrolled cognitively healthy adults aged ≥45 years to investigate cognitive ageing and its vascular determinants [24]. We also examined the associations between demographic and cardiometabolic risk factors and probable dementia across two age strata: middle-aged adults (45–59 years) and older adults (≥60 years).

## METHODS

In this cross-sectional study, we performed a secondary analysis of data from the APCAPS, an intergenerational population-based cohort established in 29 villages near Hyderabad, India (Ranga Reddy District) to investigate the long-term health outcomes consequences of the Hyderabad Nutrition Trial [25]. Our study population comprised adults aged ≥45 years who participated in the fourth APCAPS follow-up survey (2021–2023). All participants provided written informed consent to take part in the APCAPS survey, and we used de-identified data for this analysis. We confirm that our analysis aligns with the Journal of Global Health’s GRABDROP guidelines (Table S1 in the **Online Supplementary Document**) [26].

### Sociodemographic and economic variables

Interviewers administered questionnaires to collect data on sociodemographic characteristics (age, sex, family structure, marital status) and economic indicators (education, occupation, household assets). For the sociodemographic characteristics, we stratified participants into two age groups: middle-aged adults (45–59 years) and older adults (≥60 years), in alignment with the threshold adopted in major Indian dementia research, including the LASI [2]. We categorised marital status into three groups: married, widowed, and single/divorced. Because dementia status and comorbidity data were missing for all five single/divorced participants, we excluded them and analysed marital status as a binary variable (married *vs*. widowed). We otherwise classified family structure as either nuclear or joint, whereby a nuclear family comprised individuals living alone, couples without children, or parents residing with their children as a separate household unit, and a joint family referred to a multi-generational household where two or more related adult generations, such as grandparents, parents, or married siblings, lived together and share resources. We included family structure as a covariate based on the hypothesis that joint, multi-generational households confer cognitive protection. In the rural Indian context, joint families traditionally provide continuous social interaction, pooled caregiving resources, and multi-generational cognitive engagement. These mechanisms theoretically bolster cognitive reserve and protect against social isolation. Conversely, the demographic shift toward nuclear families often correlates with increased social isolation and reduced cognitive stimulation for older adults [27,28].

In terms of socioeconomic indicators, we classified educational attainment as a binary variable (illiterate *vs*. literate), combining informal literacy, primary, and secondary education into a single ‘literate’ category. This classification was based on the distribution of educational attainment in our rural sample and ensured adequate statistical power, while addressing the practical challenges of distinguishing informal literacy from minimal formal schooling in this setting. We categorised occupational status into four groups: unskilled labour (housework and unskilled manual labour); skilled labour (clerical/administrative work, trade, and manufacturing); non-working (unemployed/retired); and agriculture or animal rearing. Finally, we calculated a household asset score using 24 binary variables covering basic amenities, appliances, vehicles, furniture, property, and valuables. We also included indicators for construction materials, lighting, cooking fuel, water source, and toilet facilities, where a value of 1 represented wealthier households. Following the methodology of previous studies, we divided the final scores into tertiles (low, medium, and high) for socioeconomic stratification [29–31].

### Cardiometabolic diseases

We collected data on cardiometabolic diseases during the fourth APCAPS follow-up (2022–2023) using validated diagnostic methods described in detail in the study protocol [25]. We classified participants as having diabetes if they reported a previous diagnosis made by a physician or met biochemical criteria. We collected fasting venous blood samples, and measured plasma glucose on the day of collection using the glucose oxidase–peroxidase method (Randox Laboratories, Crumlin, Northern Ireland) at the central laboratory of the Indian Council of Medical Research–National Institute of Nutrition, Hyderabad, India. We defined diabetes as a fasting blood glucose ≥126 mg/dL among participants who confirmed fasting since the previous day, or a blood glucose level >200 mg/dL among non-fasting participants and those with missing fasting information. We excluded participants with missing data for both self-reported diagnosis and biochemical measurement from the diabetes-specific analysis.

We classified participants as having hypertension if they reported a previous diagnosis made by a physician or if the mean of their second and third brachial blood pressure readings was at least 140 mm Hg systolic or at least 90 mm Hg diastolic. We measured blood pressure in the right arm with participants seated and after they had rested for five minutes, taking three readings with validated automated oscillometric devices, either the Omron HEM-7121 (Omron Healthcare Co., Ltd., Kyoto, Japan) ir the Uscom BP+ (Uscom Ltd., Sydney, Australia).

We assessed cardiac disease using either of two measures. The first was a positive screen for coronary heart disease on the World Health Organization Rose Angina Questionnaire [32], which we classified as definite angina according to standard criteria. The second was objective evidence of left ventricular systolic dysfunction, defined as a left ventricular ejection fraction of 50% or less on two-dimensional echocardiography. We excluded participants with missing data for both the questionnaire and echocardiographic assessments. This composite definition allowed us to capture clinically significant cardiac disease through the two complementary measures available in APCAPS: symptomatic ischaemia and objective systolic dysfunction. Although these conditions are clinically distinct, both reflect systemic cardiovascular pathology and are recognised contributors to cerebrovascular risk and cognitive decline [33]. Similarly, we identified stroke using a dual-verification approach. We classified participants as having stroke if they reported a previous diagnosis made by a physician or screened positive on the symptom-based stroke questionnaire of Gourie-Devi and colleagues [34]. This approach allowed us to capture both previously diagnosed and symptomatic cases in the community.

### Dementia screening

We screened for probable dementia using the Brief Community Screening Instrument for Dementia (BCSI-D), a tool validated for low-literacy populations in India, where sensitivity and specificity were reported to be 97.3% and 90.5%, respectively [35], and used across multiple countries, including Uganda and Ghana [36,37]. We classified participants achieving a final score of ≤4 as having ‘probable dementia’ and those scoring ≥5 as having ‘no dementia’ (Figure S1 in the **Online Supplementary Document**). The assessment has 13 items: seven cognitive items administered to the participant and six informant items administered to a close contact.

### Statistical analysis

We estimated the prevalence of probable dementia, defined using the BCSI-D criteria, across age, sex and socioeconomic strata. We report prevalence with 95% confidence intervals (CIs) and visualise it using forest plots. Probable dementia refers to participants who screened positive for dementia on the BCSI-D (a final score of 4 or less). Our prevalence estimates therefore reflect positive screening results, not clinically confirmed diagnoses. To compare participant characteristics between age groups (45–59 *vs*. ≥60 years), we tested the following hypotheses for each variable (for example, sex, occupation, or probable dementia status). The null hypothesis (H0) was that there is no association between age group and the variable. The alternative hypothesis (H1) was that there is a significant association between age group and the variable. A two-sided *P*-value <0.05 was considered statistically significant.

We used logistic regression to examine associations between probable dementia and sociodemographic, socioeconomic, and cardiometabolic exposures. We first fitted univariable models to estimate unadjusted odds ratios (ORs). We then fitted a multivariable model to estimate adjusted odds ratios (aORs) and identify independent associations. Because we observed 111 cases of probable dementia, we assessed model diagnostics before finalising the multivariable model to ensure model stability and guard against overfitting. We evaluated multicollinearity using the variance inflation factor, applying a conservative threshold of <5. We assessed calibration with the Hosmer–Lemeshow test (chi-squared test = 8.57, df = 8, *P* = 0.38), which showed no evidence of poor fit. Because we aimed to estimate associations rather than build a prediction model, we did not seek to optimise predictive performance. We report the results of this modelling using ORs and aORs with 95% CIs.

We handled missing data using complete-case analysis. Overall, data were missing for 16 of 2,196 participants (0.73%) only, and no key covariates showed evidence of systematic missingness (Tables S3 and S4 in the **Online Supplementary Document**). These patterns were broadly consistent with a missing at random assumption; therefore, we did not perform multiple imputation. We conducted all statistical analyses in *R*, version 4.1.0 (R Core Team, Vienna, Austria).

## RESULTS

We analysed data from 2,196 adults aged ≥45 years out of the 4,478 participants in the fourth APCAPS follow-up. We excluded 33 participants – 10 because of very small subgroup sizes and 23 because of missing data – resulting in a final analytical sample of 2,163 individuals (**Figure 1**). Of these, 1,554 participants (72%) were illiterate and 609 (28%) were literate. The overall prevalence of probable dementia was 5.1% (**Table 1**), increasing from 3.4% in the 45–59 age group to 7.4% in the ≥60 age group (*P* < 0.001). Compared with the 45–59 age group, the ≥60 age group had a higher proportion of males (64% *vs*. 25%) and of unemployed or retired individuals (22% *vs*. 5.3%), while there were no major differences in terms of education and marital status.

**Figure 1:**
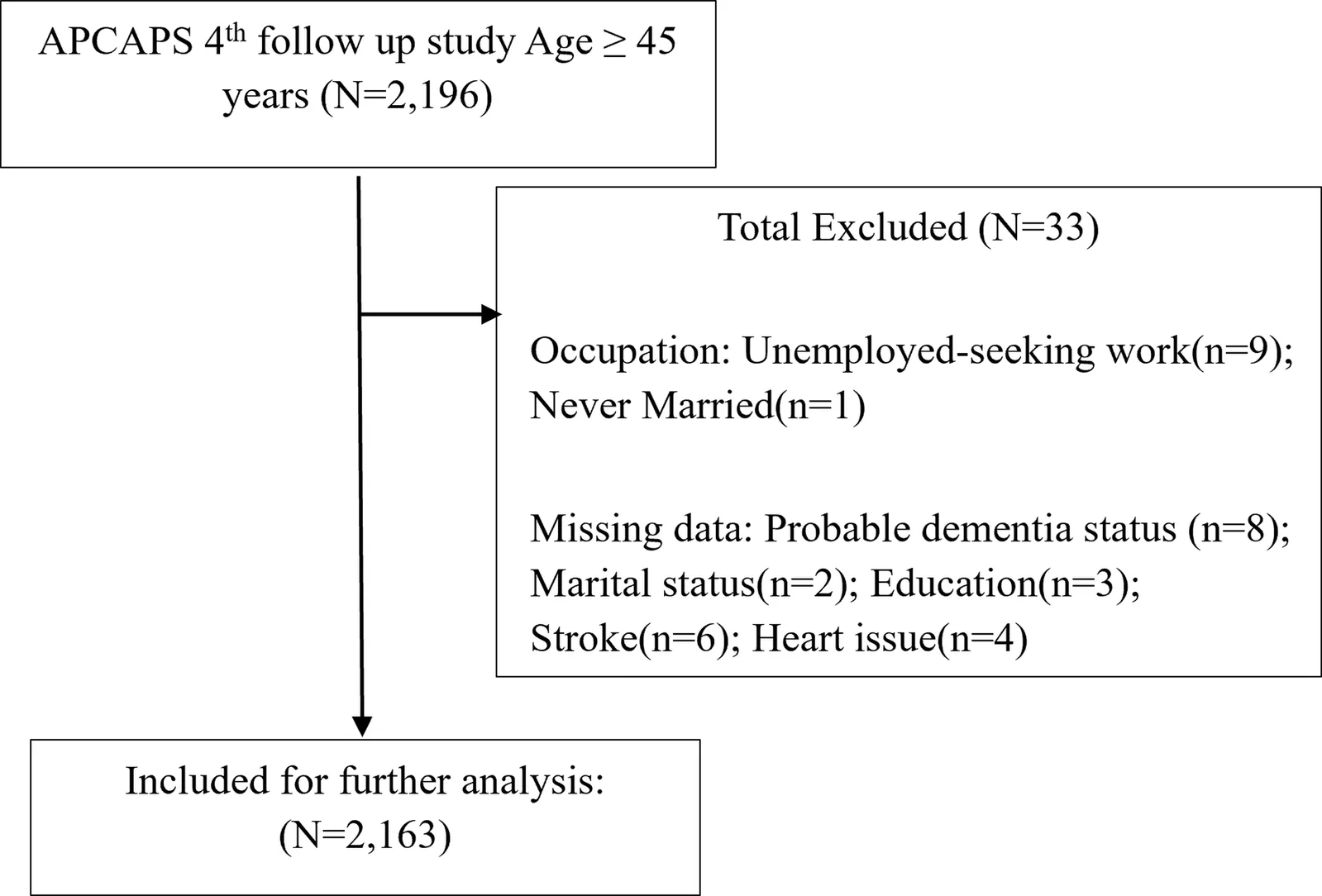
APCAPS data flow.

**Table 1:** Sociodemographic and clinical characteristics of the APCAPS study population stratified by age group (n = 2,163), n (%)

	Total	45–59 years (n = 1,234)	≥60 years (n = 929)	*P*-value*	
**Sex**				<0.001	
Female	1,258 (58.2)	922 (74.7)	336 (36.2)		
Male	905 (41.8)	312 (25.3)	593 (63.8)		
**Family size**				0.037	
Joint family	552 (25.5)	294 (23.8)	258 (27.8)		
Nuclear family	1611 (74.5)	940 (76.2)	671 (72.2)		
**Marital status**				0.342	
Married	1679 (77.6)	967 (78.4)	712 (76.6)		
Widowed	484 (22.4)	267 (21.6)	217 (23.4)		
**Education**				0.890	
Illiterate	1554 (71.8)	888 (72.0)	666 (71.7)		
Literate	609 (28.2)	346 (28.0)	263 (28.3)		
**Occupation**				<0.001	
Agriculture/animal rearing	742 (34.3)	432 (35.0)	310 (33.4)		
Skilled labour	365 (16.9)	245 (19.9)	120 (12.9)		
Unskilled labour	786 (36.3)	492 (39.9)	294 (31.6)		
Unemployed/retired	270 (12.5)	65 (5.3)	205 (22.1)		
**Assets category**				0.024	
Low	915 (42.3)	493 (40.0)	422 (45.4)		
Medium	599 (27.7)	347 (28.1)	252 (27.1)		
High	649 (30.0)	394 (31.9)	255 (27.4)		
**Health condition†**					
Diabetes	308 (14.2)	185 (15.0)	123 (13.2)	0.248	
High BP	630 (29.1)	311 (25.2)	319 (34.3)	<0.001	
Cardiovascular issues	65 (3.0)	33 (2.7)	32 (3.4)	0.299	
Stroke	54 (2.5)	22 (1.8)	32 (3.4)	0.014	
**Probable dementia status**				<0.001	
No dementia	2,052 (94.9)	1,192 (96.6)	860 (92.6)		
Dementia	111 (5.1)	42 (3.4)	69 (7.4)		

*Pearson’s chi-squared test.

†Health conditions were assessed using screening protocols, rather than diagnostic confirmation.

### Probable dementia prevalence

In the 45–59-year-old age group, probable dementia was most common in adults who were unemployed or retired (9.2%) and had a history of stroke (9.1%). Within this group, probable dementia prevalence was higher among females (3.7%) than males (2.6%) and nearly double in illiterate participants (3.9%) compared to their literate counterparts (2.0%) (**Figure 2**). Conversely, in the ≥60-year-old age group (**Figure 3**), the prevalence of probable dementia was higher among males (8.6%) than females (5.4%) and was notably high among unemployed or retired individuals (12.7%). The condition was also particularly prevalent among participants with a history of cardiovascular disease in this age group, with a prevalence of 28.1% among those with a history of stroke and 9.4% among those with heart-related issues.

**Figure 2:**
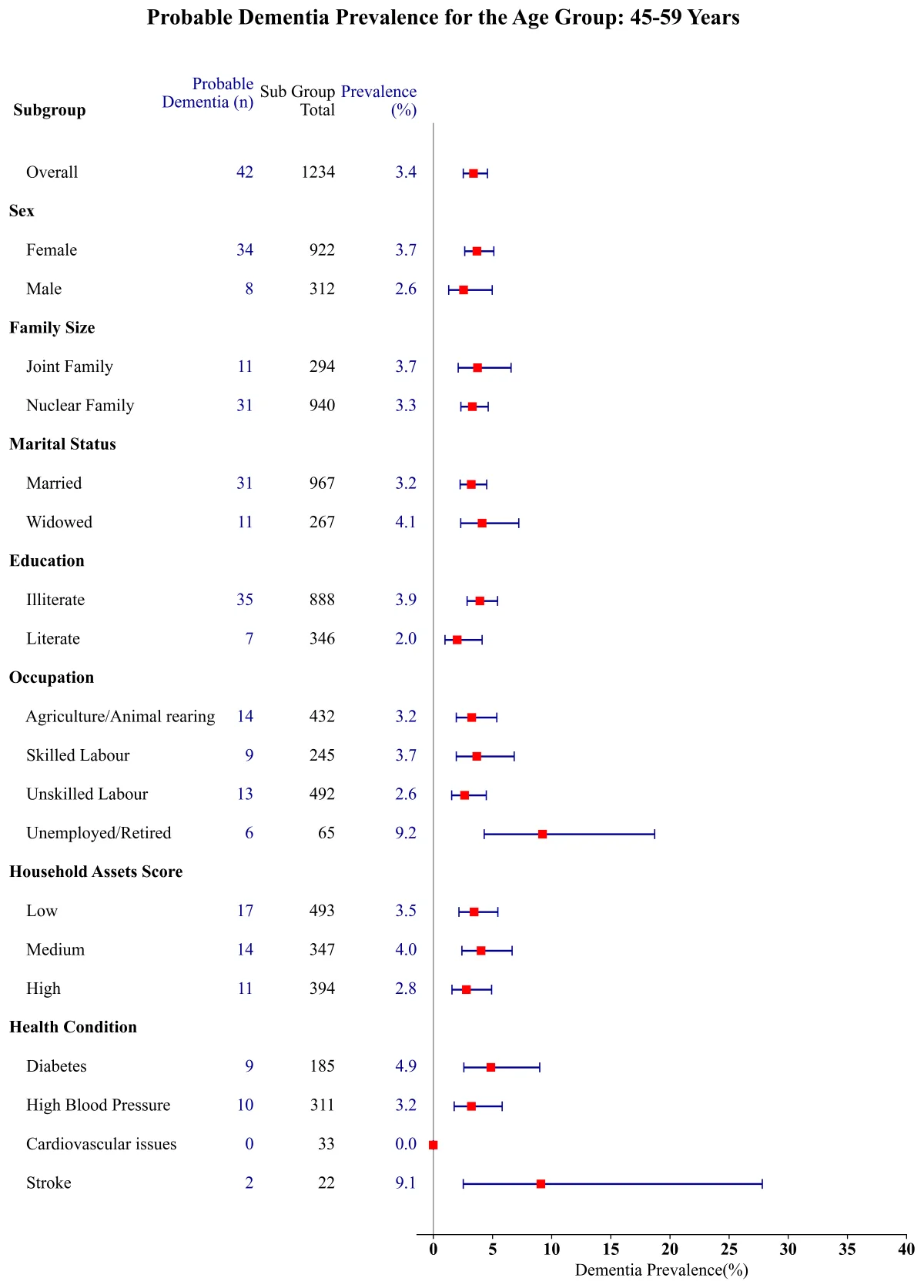
Probable dementia prevalence among 45–59-year-olds.

**Figure 3:**
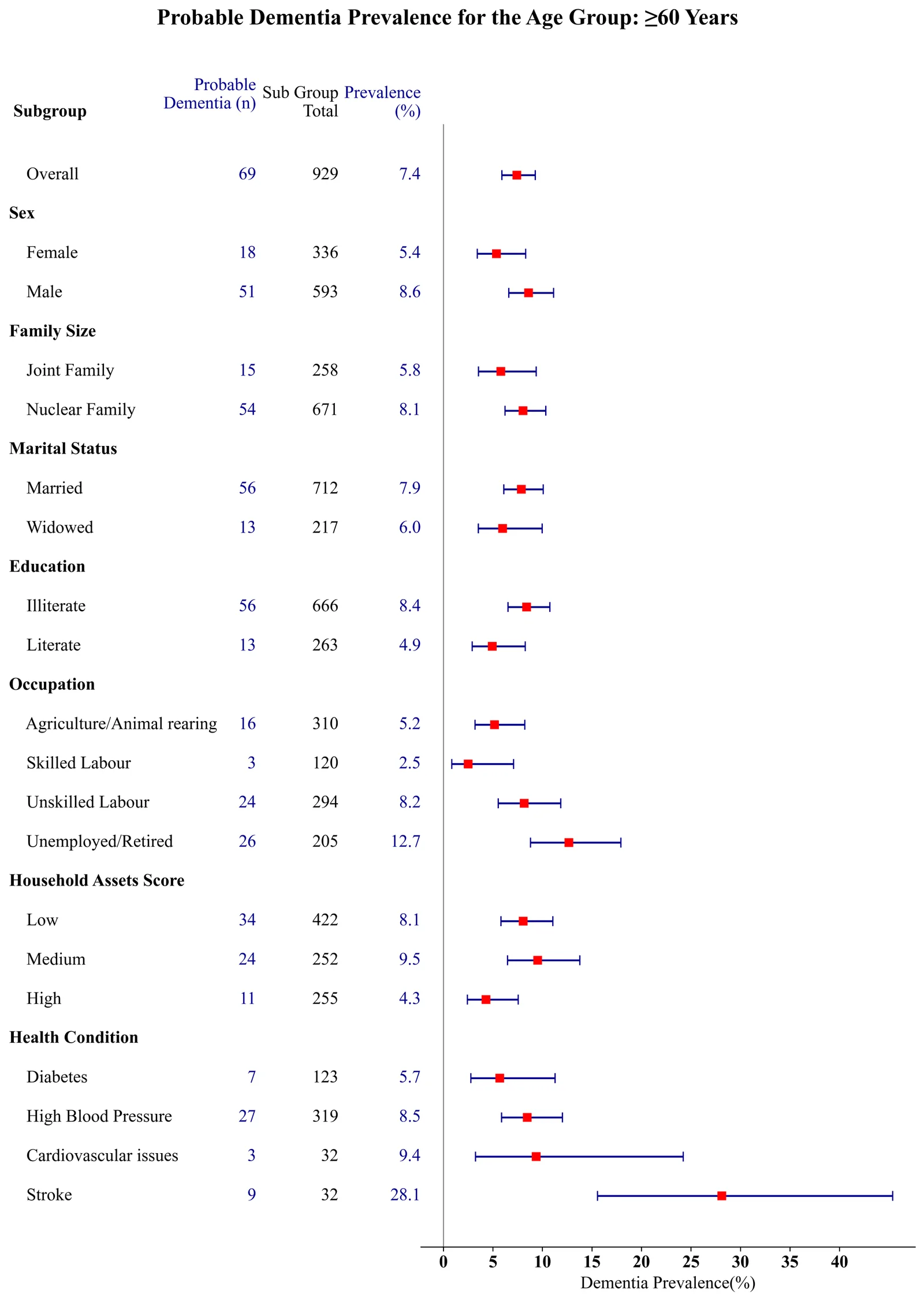
Probable dementia prevalence for ≥60-year-olds.

### Associations with probable dementia

 Model diagnostics confirmed the stability of the multivariable model: all variance inflation factors were below the conservative threshold of 5 (maximum equivalent variance inflation factor = 1.57). Age ≥60 years was associated with higher odds of probable dementia compared with age 45–59 years (aOR = 1.69; 95% CI = 1.07–2.67, *P* = 0.024). Being unemployed or retired was associated with higher odds of probable dementia compared with working in agriculture or animal rearing (aOR = 2.23; 95% CI = 1.26–3.95, *P* = 0.006). Literacy was associated with lower odds of probable dementia compared with illiteracy (aOR = 0.54; 95% CI = 0.31–0.89, *P* = 0.021). After adjustment, probable dementia was not significantly associated with sex, marital status, family size, household asset score, or the other occupational categories (T**Table 2**).

**Table 2:** Prevalence of probable dementia and associations with sociodemographic characteristics and cardiometabolic diseases (n = 2,163)

	No dementia (n = 2052), n (%)	Probable dementia (n = 111), n (%)	OR (95% CI)	*P*-value	aOR (95% CI)	*P*-value	
**Age**							
45–59	1,192 (96.6)	42 (3.4)	ref		ref		
≥60	860 (92.6)	69 (7.4)	2.28 (1.54–3.40)	<0.001	1.69 (1.07–2.67)	0.024	
Sex							
Female	1,206 (95.9)	52 (4.1)	ref		ref		
Male	846 (93.5)	59 (6.5)	1.62 (1.10–2.38)	0.014	1.32 (0.81–2.15)	0.269	
**Family size**							
Joint family	526 (95.3)	26 (4.7)					
Nuclear family	1,526 (94.7)	85 (5.3)	1.13 (0.73–1.80)	0.603	1.17 (0.75–1.90)	0.498	
**Marital status**							
Married	1,592 (94.8)	87 (5.2)	ref		ref		
Widowed	460 (95.0)	24 (5.0)	0.95 (0.59–1.49)	0.845	0.88 (0.51–1.46)	0.624	
**Education**							
Illiterate	1,463 (94.1)	91 (5.9)	ref		ref		
Literate	589 (96.7)	20 (3.3)	0.55 (0.32–0.88)	0.016	0.54 (0.31–0.89)	0.021	
**Occupation**							
Agriculture/animal rearing	712 (96.0)	30 (4.0)	ref		ref		
Skilled labour	353 (96.7)	12 (3.3)	0.81 (0.39–1.56)	0.537	0.92 (0.44–1.79)	0.803	
Unskilled labour	749 (95.3)	37 (4.7)	1.17 (0.72–1.93,)	0.527	1.20 (0.72–2.03)	0.483	
Unemployed/retired	238 (88.1)	32 (11.9)	3.19 (1.90–5.38)	<0.001	2.23 (1.26–3.95)	0.006	
**Asset score**							
Low	864 (94.4)	51 (5.6)	ref		ref		
Medium	561 (93.7)	38 (6.3)	1.15 (0.74–1.77)	0.534	1.20 (0.76–1.87)	0.428	
High	627 (96.6)	22 (3.4)	0.59 (0.35–0.98)	0.046	0.64 (0.37–1.08)	0.106	
**Diabetes**							
No	1,760 (94.9)	95 (5.1)	ref		ref		
Yes	292 (94.8)	16 (5.2)	1.02 (0.57–1.70)	0.957	1.13 (0.62–1.95)	0.678	
**High BP**							
No	1,459 (95.2)	74 (4.8)	ref		ref		
Yes	593 (94.1)	37 (5.9)	1.23 (0.81–1.83)	0.317	0.94 (0.60–1.47)	0.803	
**Cardiovascular issues**							
No	1,990 (94.9)	108 (5.1)	ref		ref		
Yes	62 (95.4)	3 (4.6)	0.89 (0.22–2.46,)	0.848	0.74 (0.17–2.16)	0.635	
**Stroke**							
No	2,009 (95.3)	100 (4.7)	ref		ref		
Yes	43 (79.6)	11 (20.4)	5.14 (2.46–9.93)	<0.001	3.77 (1.67–7.94)	0.001	

aOR – adjusted odds ratio, BP – blood pressure, CI – confidence interval, OR – odds ratio, ref – reference

A history of stroke showed the strongest association with probable dementia (aOR = 3.77; 95% CI = 1.67–7.94, *P* = 0.001). After adjustment, diabetes, high blood pressure, and cardiovascular disease were not significantly associated with probable dementia (**Table 2**).

## DISCUSSION

Using data from the APCAPS community cohort, we estimated the prevalence of probable dementia in a rural, transitional Indian population to be 3.4% (95% CI = 2.6–4.6) in adults aged 45–59 years and 7.4% (95% CI = 6.0–9.4) in those aged ≥60 years. Low educational attainment and unemployment/retirement emerged as the strongest sociodemographic determinants of probable dementia, with a history of stroke was a robust predictor (aOR = 3.8) being a robust predictor of the condition among cardiometabolic diseases. These findings show that probable dementia, identified by positive dementia screen, extends into middle age, exacerbated by stroke and low education in rural populations.

Our estimate of the prevalence of probable dementia being 7.4% in the ≥60 age group aligns closely with the 7.4% national average reported by the LASI, though it is slightly lower than the LASI estimate of 8.4% for the rural population [2]. This supports the evidence stemming from existing studies that rural populations bear a substantial burden of cognitive impairment, likely exacerbated by lower cognitive reserve and limited access to healthcare [38,39].

Evidence on the prevalence of dementia among people aged <60 years in India is limited, because major population-based epidemiological studies, such as those of the 10/66 Dementia Research Group, typically exclude this age group [2,40]. In our study, 3.4% of adults aged 45–59 years screened positive for probable dementia. This estimate is similar to that of a 2011 study in rural Uttar Pradesh, which reported a positive dementia screen prevalence of 2.6% in adults aged 50–59 years [11]. We hypothesise that the small difference between the two estimates reflects improved case ascertainment and the changing epidemiological landscape since 2011. Furthermore, our findings suggest a higher potential burden in India compared to high-income countries, where dementia at younger ages is rare (<1%) [21].

While these findings are significant, the estimated prevalence of 3.4% for probable dementia in the 45–59 age group warrants cautious interpretation. With 72% of the cohort being illiterate and cognitive screening tasks being highly sensitive to educational attainment, some proportion of positive screens may reflect educational disadvantage or cultural unfamiliarity with formal testing, rather than true neurodegeneration [41]. Although the BCSI-D was specifically developed for low-literacy Indian populations and has demonstrated good diagnostic performance residual misclassification related to educational and cultural factors cannot be fully excluded.

Literacy was independently associated with lower odds of probable dementia (aOR = 0.54; 95% CI = 0.31–0.89), consistent with the cognitive reserve hypothesis [42]. This is corroborated by a recent meta-analysis corroborates this protective effect, demonstrating that cognitive reserve accumulated across the life course, meaning in both early life (hazard ratio (HR) = 0.8; 95% CI = 0.8–0.9) and late life (HR = 0.8; 95% CI = 0.8–0.9), confers significant protection against dementia [42,43].

While being unemployed or retired was associated with higher odds of probable dementia (aOR = 2.23; 95% CI = 1.26–3.95), the cross-sectional design of our study precludes us from ascertaining a causal relationship [40,42]. Reverse causation thus also remains possible, as longitudinal evidence has demonstrated that subtle cognitive deficits commonly precede workforce exit by several years, a pathway amplified in rural informal economies, where leaving work more readily reflects functional decline than a normative life transition [44,45].

Regarding cardiometabolic diseases, the strong association with stroke and probable dementia (aOR = 3.8) is consistent with the well-established contribution of cerebrovascular disease to cognitive impairment [13,46]. The lack of statistically significant associations between hypertension or diabetes and probable dementia in our study contrasts with the findings from midlife cohort studies conducted in high-income countries [47,48]. This discrepancy could partly be attributable to survival bias, where individuals with severe vascular disease die prior to screening, as well as the phenomenon of declining blood pressure during the prodromal phase of dementia, which can obscure associations in cross-sectional analyses of older adults [49,50]. In addition, the relatively small number of probable dementia cases may have limited our ability to detect modest associations, consistent with recent findings from the LASI in India, which reported only a modest association between hypertension and dementia (relative risk = 1.4) and no significant association for diabetes [1]. Residual confounding and potential overadjustment may also have attenuated the observed associations. Therefore, these null findings should be interpreted with caution.

Extrapolating the 3.4% positive screening rate in the 45–59 age group to the wider population of India would mean that approximately 8.1 million individuals nationally may warrant further diagnostic evaluation for dementia (Table S5 in the **Online Supplementary Document**). These figures should be interpreted as illustrative projections that highlight the potential scale of the public health challenge presented by the condition, rather than precise national estimates, given socioeconomic and healthcare system heterogeneity between this rural southern Indian cohort and the broader national population. This projected burden highlights two evidence-informed priorities. First, strengthening policies targeting modifiable vascular and metabolic risk factors may be particularly important among stroke survivors, who represent a high-risk group [51]. Second, given that informal care constitutes the majority of dementia-related expenditure in India and formal long-term care infrastructure remains limited, strengthening caregiver support programmes is an important component of dementia care planning [51–53]. However, as we derive our findings from a cross-sectional screening study, longitudinal studies with diagnostic confirmation are warranted before population-level screening from the age of 45 years is considered.

### Strengths and limitations

This study’s primary strengths include its community-based design focused on a rural setting in India, age stratification that allowed for the comparison of younger and older adults, the comprehensive measurement of a range of sociodemographic and cardiometabolic factors, and the use of the BCSI-D, a screening tool validated for low-literacy populations. However, several limitations must be acknowledged. First, the cross-sectional design precludes causal inference, particularly regarding the directionality of the associations of probable dementia with unemployment or retirement and with cardiometabolic diseases. Second, because the analysis relies on the fourth follow-up of the APCAPS cohort, it is highly susceptible to selection bias from cumulative attrition. Differential survival, out-migration, and non-response may have created a ‘healthy survivor’, effect, where individuals with advanced cognitive decline or fatal cardiometabolic comorbidities are underrepresented. Consequently, this survivor bias likely attenuated the observed associations between cardiometabolic risks and probable dementia and may have led to an underestimation of the true prevalence of positive dementia screens. Third, the BCSI-D is a brief, memory-focused screening tool that identifies the risk of probable dementia, rather than provides a definitive clinical diagnosis or ascertaining specific dementia subtypes [35]. Consequently, although stroke was strongly associated with probable dementia, the instrument cannot distinguish vascular dementia from persistent post-stroke cognitive impairment or other dementia subtypes [54]. Therefore, the underlying pathological mechanism of the observed cognitive impairment cannot be determined from the present study. Additionally, the assessment of cognitive decline in a population with 72% illiteracy may have reduced our ability to accurately differentiate between mild cognitive impairment and dementia [41]. Fourth, as this was a study conducted in a single rural setting in southern India, its findings cannot be fully generalised to northern states, which have different cultural and socioeconomic profiles, or to other contexts. Finally, although the multivariable model demonstrated an acceptable events-per-variable ratio and no evidence of problematic multicollinearity, the relatively small number of probable dementia cases may have limited the precision of some effect estimates and reduced our ability to detect modest associations.

## CONCLUSIONS

In this cross-sectional analysis of data from the fourth follow-up of the APCAPS cohort, we observed a high prevalence of positive dementia screens in rural India, indicating an at-risk population that extends into middle age. The rapid ageing of India’s population is expected to lead to an increase in the number of people living with dementia, while limited access to healthcare in rural areas and low education levels in these settings will likely exacerbate this issue. Left unaddressed, this burden could impose severe social, economic, and caregiving strains on the country. However, as our study cross-sectional and relies on screening rather than clinical diagnosis, these results should inform research priorities, rather than immediate policy action.

## Additional material


Online Supplementary Document

## Data Availability

**Data availability:** Deidentified data and metadata are available upon reasonable request and following approval by the APCAPS executive committee. Data collection instruments and cohort details are accessible at the project website(www.lshtm.ac.uk/research/centres-projects-groups/apcaps). Please contact the study team at apcaps.crf@gmail.com to initiate a collaboration request.
