# Supplementary material for: Prevalence and determinants of probable dementia in rural India: a cross-sectional study in the APCAPS cohort
Source: J Glob Health. 2026 Oct 19;16:04339. doi: 10.7189/jogh.16.04339 (PMC13679355; doi:10.7189/jogh.16.04339)

Supplement to: Basa JR, Pinyopornpanish K, Mallinson PAC, Inkeaw P, Kulprachakarn K, Lieber J, Banjara SK, Mahajan H, Dasi T, Bhogadi S, Addanki S, Iyer G, Shah AS, Kurmi O, Kamalakannan S, Kinra S, Angkurawaranon C. Prevalence and determinants of probable dementia in rural India: a cross-sectional study in the APCAPS cohort. J Glob Health. 2026;16:04339.

Table S1. Adherence to JoGH's GRABDROP guidelines items.

**JoGH guideline items**
1. Please list all papers published by each co-author in previous 3 years that were based on secondary analysis of a big data repository
[1] N. Birk, B. Kulkarni, S. Bhogadi, A. Aggarwal, G. K. Walia, V. Gupta et al., "Machine learning-based equations for improved body composition estimation in Indian adults," PLOS Digital Health, vol. 4, no. 6, p. e0000671, 2025, doi: 10.1371/journal.pdig.0000671.<br><br>[2] H. Mahajan, P. A. C. Mallinson, J. Lieber, S. Bhogadi, S. K. Banjara, V. S. Reddy et al., "The Association of Total Meat Intake with Cardio-Metabolic Disease Risk Factors and Measures of Sub-Clinical Atherosclerosis in an Urbanising Community of Southern India: A Cross-Sectional Analysis for the APCAPS Cohort," Nutrients, vol. 16, no. 5, p. 746, 2024. [Online]. Available: https://www.mdpi.com/2072-6643/16/5/746.<br><br>[3] H. Mahajan, P. A. C. Mallinson, J. Lieber, S. Bhogadi, S. K. Banjara, A. Shah et al., "Comparison of laboratory-based and non-laboratory-based WHO and GLOBORISK CVD risk scores: A cross-sectional analysis of the APCAPS cohort," (in eng), PLoS One, vol. 21, no. 2, p. e0342471, 2026, doi: 10.1371/journal.pone.0342471.<br><br>[4] I. Rudan, P. Song, D. Adeloye, and H. Campbell, "Journal of Global Health's Guidelines for Reporting Analyses of Big Data Repositories Open to the Public (GRABDROP): preventing 'paper mills', duplicate publications, misuse of statistical inference, and inappropriate use of artificial intelligence," Journal of Global Health, vol. 15, p. 01004, 2025.<br><br>[5] C. Sharma, A. Goncalves Soares, B. Kulkarni, N. M. Byrne, S. Bhogadi, P. A. C. Mallinson et al., "Life-course trajectories of cardiovascular disease risk factors in rural India: Andhra Pradesh Children and Parents Study (APCAPS) 2003–2023," International Journal of Epidemiology, vol. 55, no. 4, 2026, doi: 10.1093/ije/dyag080.<br><br>[6] T. Yotruangsri, P. Aramrat, C. Aramrat, S. Pliannuom, C. Angkurawaranon, K. Pinyopornpanish. "CNS-active medication use and adverse health outcomes among Thai older adults: a population-based retrospective study," Sci Rep, vol. 16, no. 1, 2026, doi: 10.1038/s41598-026-37555-2.
2. Please explain the key elements of your study design and the use of the available datasets that make your study an original scientific contribution
First, to the best of our knowledge, this study provides one of the first population-based estimates of screening-based probable dementia prevalence among adults aged 45–59 years in rural South India, addressing an evidence gap identified in recent analyses of Indian ageing cohorts, including LASI (2023).

Second, the study leverages data from the APCAPS intergenerational cohort, enabling the examination of midlife and older-age cognitive outcomes within a long-standing population-based framework.<br><br>Third, we simultaneously modelled socioeconomic and cardiometabolic determinants of probable dementia within the same analytical framework, providing a more comprehensive assessment of risk factors in this setting.<br><br>Forth, we observed a strong independent association between stroke and probable dementia (aOR 3.8), highlighting a potentially important vascular contribution to cognitive impairment in this population, although causal pathways cannot be inferred from the cross-sectional design.
3. Please list all publications that addressed similar research questions in the same dataset and indicate where you cited them in your paper
This is the first study, using the APCAPS 4th follow up study data and there is no research papers published
4. Please explain how you addressed multiple testing through an appropriately rigorous statistical threshold and indicate this in the methods section
We did not adjust for multiple testing because analyses were hypothesis-driven and should be taken as hypothesis generating.
5. Please declare to what extent have AI chatbots been used in developing your paper and to which parts of the paper did they contribute
AI chatbots (e.g., Microsoft Copilot) were employed to refine grammatical structure and enhance sentence coherence across all sections of the manuscript.

Table S2. Brief Community Screening for Dementia Questionnaire

Test Type	Qn No	Question/Item
**Cognitive**	1	How do you use a hammer?
**Cognitive**	2	What is this body part called? (Show: elbow)
**Cognitive**	3	Can you point to the window, and then to the door?
**Cognitive**	4	Where is the nearest store located?
**Cognitive**	5	What season is it right now?
**Cognitive**	6	What day of the week is it today?
**Cognitive**	7	(After delay) Can you recall the three words I mentioned earlier?
**Informant**	1	Does she/he often forget where she/he put things?
**Informant**	2	Have you noticed a general decline in her/his mental functioning?
**Informant**	3	Have you noticed any change in her/his ability to think and reason?
**Informant**	4	Does she/he sometimes forget what happened the day before?
**Informant**	5	Does she/he sometimes forget where she/he is?
**Informant**	6	Does she/he have any difficulty dressing?

Table S3. Missing Data Summary – APCAPS Wave 4 (N = 2,196)

Variable	N Missing	% Missing	N Complete
**Household structure (ha1q6_1household)**	4	0.18%	2,192
**Marital status**	6	0.27%	2,190
**Education**	3	0.14%	2,193
**Occupation**	7	0.32%	2,189
**Diagnosed diabetes**	6	0.27%	2,190
**Diagnosed heart disease**	6	0.27%	2,190
**Diagnosed stroke**	11	0.50%	2,185
**Diagnosed hypertension**	6	0.27%	2,190
**Total excluded (any missing)**	**16**	**0.73%**	**2,180**

Note: Some participants had missing data on more than one variable; total excluded (16) is the count of participants with at least one missing value across all listed variables.

Table S4. MCAR Chi-Square Independence Test Results

Binary missingness indicators were constructed for each variable with missing data and tested for independence against all fully observed covariates (age group, gender, household asset score, continuous BCSI-D dementia score, informant score, and binary dementia classification) using Pearson's chi-square tests. Under the null hypothesis of Missing Completely at Random (MCAR), the missingness indicator should be statistically independent of all observed covariates ($p \geq 0.05$).

Missing Variable	Covariate	χ^2	df	p-value	MCAR?
**Household structure**	Age group	0.000	1	1.000	✓
**Household structure**	Gender	0.000	1	1.000	✓
**Household structure**	Household assets	4.425	2	0.109	✓
**Household structure**	BCSI-D score (continuous)	77.648	9	<0.001	✗ *
**Household structure**	Informant score	0.252	6	1.000	✓
**Household structure**	Dementia classification (binary)	0.435	1	0.510	✓
**Marital status**	Age group	0.779	1	0.377	✓
**Marital status**	Gender	0.695	1	0.405	✓
**Marital status**	Household assets	1.442	2	0.486	✓
**Marital status**	BCSI-D score (continuous)	728.983	9	<0.001	✗ *
**Marital status**	Informant score	0.379	6	0.999	✓

**Marital status**	Dementia classification (binary)	0.000	1	1.000	✓
**Education**	Age group	0.064	1	0.801	✓
**Education**	Gender	0.084	1	0.772	✓
**Education**	Household assets	2.663	2	0.264	✓
**Education**	BCSI-D score (continuous)	1.106	9	0.999	✓
**Education**	Informant score	0.189	6	1.000	✓
**Education**	Dementia classification (binary)	0.000	1	1.000	✓
**Occupation**	Age group	1.311	1	0.252	✓
**Occupation**	Gender	1.193	1	0.275	✓
**Occupation**	Household assets	0.808	2	0.668	✓
**Occupation**	BCSI-D score (continuous)	624.316	9	<0.001	χ *
**Occupation**	Informant score	0.442	6	0.998	✓
**Occupation**	Dementia classification (binary)	0.000	1	1.000	✓
**Diagnosed diabetes**	Age group	0.779	1	0.377	✓
**Diagnosed diabetes**	Gender	0.695	1	0.405	✓
**Diagnosed diabetes**	Household assets	1.442	2	0.486	✓
**Diagnosed diabetes**	BCSI-D score (continuous)	728.983	9	<0.001	χ *
**Diagnosed diabetes**	Informant score	0.379	6	0.999	✓

**Diagnosed diabetes**	Dementia classification (binary)	0.000	1	1.000	✓
**Diagnosed heart disease**	Age group	0.779	1	0.377	✓
**Diagnosed heart disease**	Gender	0.695	1	0.405	✓
**Diagnosed heart disease**	Household assets	1.442	2	0.486	✓
**Diagnosed heart disease**	BCSI-D score (continuous)	728.983	9	<0.001	χ *
**Diagnosed heart disease**	Informant score	0.379	6	0.999	✓
**Diagnosed heart disease**	Dementia classification (binary)	0.000	1	1.000	✓
**Diagnosed stroke**	Age group	3.842	1	0.050	χ †
**Diagnosed stroke**	Gender	1.646	1	0.199	✓
**Diagnosed stroke**	Household assets	2.298	2	0.317	✓
**Diagnosed stroke**	BCSI-D score (continuous)	621.963	9	<0.001	χ *
**Diagnosed stroke**	Informant score	0.696	6	0.995	✓
**Diagnosed stroke**	Dementia classification (binary)	0.009	1	0.923	✓
**Diagnosed hypertension**	Age group	0.779	1	0.377	✓
**Diagnosed hypertension**	Gender	0.695	1	0.405	✓

**Diagnosed hypertension**	Household assets	1.442	2	0.486	✓
**Diagnosed hypertension**	BCSI-D score (continuous)	728.983	9	<0.001	✗ *
**Diagnosed hypertension**	Informant score	0.379	6	0.999	✓
**Diagnosed hypertension**	Dementia classification (binary)	0.000	1	1.000	✓

Summary: 40 of 48 tests (83.3%) confirm MCAR ($p \geq 0.05$)

* Association confined exclusively to the continuous BCSI-D score (the model outcome), not to any sociodemographic covariate. This pattern is consistent with Missing at Random (MAR) see interpretation below.

† Diagnosed stroke × Age group: $p = 0.050$ (exactly at threshold); only 11 missing stroke records. This borderline result carries no meaningful systematic implication given the negligible volume of missing data.

Table S5. Estimated Prevalence and Absolute Burden of Probable Dementia in India (2021–23) Stratified by Age Group and Comorbid Vascular Conditions

	Age: 45-59 Years			Age: ≥60 Years		
	Estimated population (in Millions)	Estimated Probable Dementia %	Estimated population with Probable dementia (in Millions)	Estimated population (in Millions)	Estimated Probable Dementia %	Estimated population with Probable dementia (in Millions)
**India Population: 2021-23**	238[57]	3.4%	8.1	138	7.4%	10.2
**Diabetes**	44[8]	4.9%	2.2	26	5.7%	1.5
**High Blood Pressure**	95[8]	3.2%	3.0	69	8.5%	5.8
**Cardiovascular Diseases**	52[58]	0.0%	0.0	52	9.4%	4.9
**Stroke**	2[59]	9.1%	0.2	3	28.1%	0.8

Data Sources: Population denominators and health condition estimates are derived from multiple longitudinal and cross-sectional studies as cited in the table.

Probable Dementia: Defined based on brief CSI-D scores.

Estimated Population: Figures are presented in millions and rounded to the nearest decimal.

Population projections to indicate these are estimates of individuals at risk requiring further diagnostic evaluation, rather than confirmed clinical cases.

Figure S1 Brief community screening protocol for probable dementia

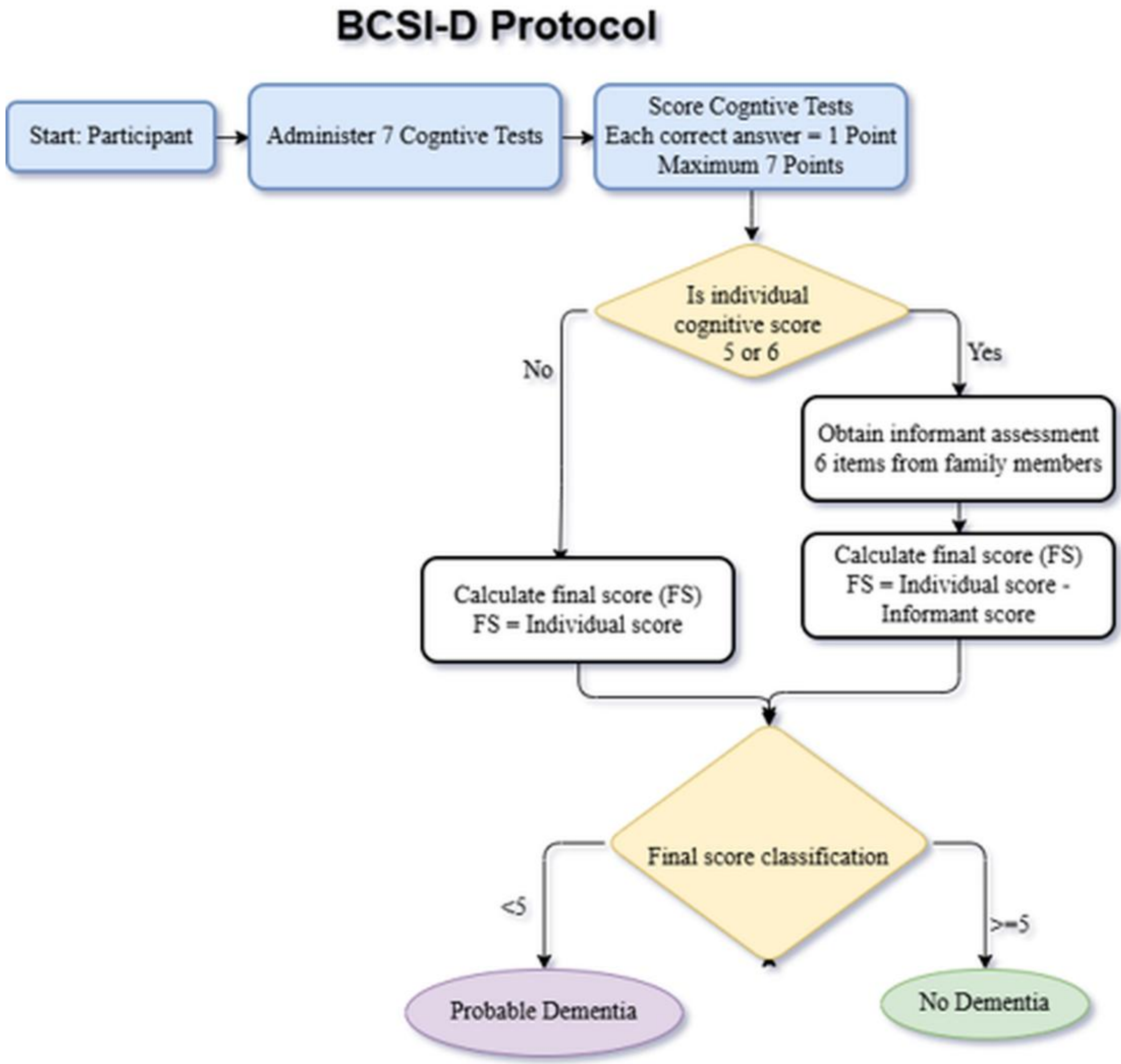

Supplement: Supplementary file 1 [file jogh-16-04339-s001.pdf]
